# Combination of Autoantibody Signature with PSA Level Enables a Highly Accurate Blood-Based Differentiation of Prostate Cancer Patients from Patients with Benign Prostatic Hyperplasia

**DOI:** 10.1371/journal.pone.0128235

**Published:** 2015-06-03

**Authors:** Petra Leidinger, Andreas Keller, Lisa Milchram, Christian Harz, Martin Hart, Angelika Werth, Hans-Peter Lenhof, Andreas Weinhäusel, Bastian Keck, Bernd Wullich, Nicole Ludwig, Eckart Meese

**Affiliations:** 1 Department of Human Genetics, Medical School, Saarland University, Building 60, 66421 Homburg/Saar, Germany; 2 Chair for Clinical Bioinformatics, Saarland University, Building E.2.1, 66123 Saarbrücken, Germany; 3 Center for Bioinformatics, Saarland University, Building E.1.1, 66123 Saarbrücken, Germany; 4 Austrian Institute of Technology GmbH, Health & Environment Department, 1220 Wien, Austria; 5 University Clinic of Urology, Friedrich-Alexander-University Erlangen-Nürnberg, 91054 Erlangen, Germany; 6 Medical Practice of Urology, 66877 Ramstein-Miesenbach, Germany; Hormel Institute, University of Minnesota, UNITED STATES

## Abstract

Although an increased level of the prostate-specific antigen can be an indication for prostate cancer, other reasons often lead to a high rate of false positive results. Therefore, an additional serological screening of autoantibodies in patients’ sera could improve the detection of prostate cancer. We performed protein macroarray screening with sera from 49 prostate cancer patients, 70 patients with benign prostatic hyperplasia and 28 healthy controls and compared the autoimmune response in those groups. We were able to distinguish prostate cancer patients from normal controls with an accuracy of 83.2%, patients with benign prostatic hyperplasia from normal controls with an accuracy of 86.0% and prostate cancer patients from patients with benign prostatic hyperplasia with an accuracy of 70.3%. Combining seroreactivity pattern with a PSA level of higher than 4.0 ng/ml this classification could be improved to an accuracy of 84.1%. For selected proteins we were able to confirm the differential expression by using luminex on 84 samples. We provide a minimally invasive serological method to reduce false positive results in detection of prostate cancer and according to PSA screening to distinguish men with prostate cancer from men with benign prostatic hyperplasia.

## Introduction

Prostate cancer is one of the most lethal cancers in men worldwide and the second most frequent cancer-related cause of death in the United States. In 2012, prostate cancer was estimated to account for more than 417,000 new cases and 92,000 cancer-related deaths in Europe [1]. Mostly, more than two-thirds of all prostate cancers are diagnosed in men aged 65 years and older.[2] Prostate cancer is often characterized by a gradual development and progress.[3] According to histological patterns of carcinoma cells the prostate cancer progress is graded by the Gleason scoring: well-differentiated carcinoma cells (Gleason score 2–4), moderately differentiated carcinoma cells (Gleason score 5–7), and poorly differentiated carcinoma cells (Gleason score 8–10).[4] Prostate cancer patients with Gleason score 8 to 10 run a more than three times higher risk of dying from prostate cancer within 10 years than patients with Gleason score 2 to 4 (8.3%).[5] Indeed, the disease is curable when it is early detected.[2]

Approximately, two-thirds of US men aged 50 and older are regularly—or at least once—screened for prostate cancer.[6] Digital rectal examination (DRE), and prostate-specific antigen (PSA) screening have become well-established methods in prostate cancer diagnostic. However, DRE requires long-time experience in cancer detection. Furthermore, DRE is not a sensitive tool for early disease. It often detects cancer at late stages.[7]

PSA, a serine protease, is an organ specific molecule produced by the prostatic epithelium. PSA tests introduced in the 1980s provide the opportunity to detect cancer without a positive DRE result. According to the US Food and Drug Administration, which allowed PSA testing as diagnostic tool in 1994 a PSA, level greater than 4 ng/ml is regarded as a critical value. A PSA level less than 4 ng/ml corresponds to normal range. PSA can be detected either as “free” (free PSA) or “bound” (PSA-ACT) form in patients´ sera.[8] Stenman and coworkers revealed that men with a high level of bound PSA run a higher risk of suffering from prostate cancer whereas the level of free PSA was shown to be lower in prostate cancer patients than in men with benign prostatic hyperplasia.[9] Partin et al. were able to detect prostate cancer with a sensitivity of 95% and a specificity of 20% by a free PSA screening.[10] Although PSA screenings decrease the mortality rate of prostate cancer patients, the screening method is inefficient and leads to limitations, which often result in a high rate of false positive results followed by unnecessary prostate biopsies [11–13]. Furthermore, older men commonly have a higher PSA level not corresponding to any prostate disease.[14] High PSA levels are also detectable in patients suffering from benign prostatic hyperplasia.[15] Hence, further screening methods with higher efficiency for cancer are necessary.

Biomarkers have developed into a necessary clinical tool. Especially, cancer markers have to fulfill several criteria. They have to have a high sensitivity and specificity, be easy to detect, economical, and significantly expressed. Current studies showed the potential of autoantibody screening in different types of cancer, e.g., lung cancer, breast carcinoma, ovarian tumor, and meningioma.[16–19] Autoantibodies reveal the possibility of early detection and successive therapy. The onset of autoantibody also allows a more detailed look into molecular processes in early disease development. We recently described a blood testing method using autoantibodies to identify lung cancer with a sensitivity of 97.9% and a specificity of 97.0%.[20] Moreover, Wang and co-workers established a phage-protein microarray to identify autoantibodies in prostate cancer.[21] In their study, they detect prostate cancer with a specificity of 88.2% and a sensitivity of 81.6%. Indeed, the study also detected several proteins with less homology to known proteins.

In the present study, we describe a protein macroarray screening to detect autoantibodies in prostate cancer patients`sera. In total, we analyzed 136 blood samples: 48 samples of prostate cancer patients, 60 samples of benign prostatic hyperplasia, and 28 blood samples of healthy individuals. We show that the screening allows the discrimination of sera of prostate cancer patients, and controls. We also show that immunogenic antigens can be helpful to differentiate prostate cancer patients from benign prostatic hyperplasia patients. Additionally, patients suffering from benign prostatic hyperplasia can be separated from controls. Furthermore, we focused on the following question: Can the combination of PSA and autoantibody testing improve the sensitivity and the specificity for the diagnosis of prostate cancer?

PSA testing as a single diagnostic method often leads to false positive results. New biomarker may provide an opportunity to enhance the sensitivity and the specificity for prostate cancer. Specific autoantibody screening along with the PSA test may be a useful diagnostic tool to detect prostate cancer at early stage and to reduce prostate cancer-related mortality. Moreover, the identification of novel immunogenic antigens may be a first step towards the development of new therapies.

## Materials and Methods

### Study population

Blood samples of prostate cancer patients and patients with benign prostatic hyperplasia were obtained from the Department of Urology, Saarland University Hospital. Blood samples from healthy donors were obtained from the Department of Hemostaseology and Transfusion Medicine, Saarland University Hospital. Serum was prepared from serum monovettes and stored in 2 ml aliquots at -70°C. All samples were obtained with patients´ written informed consent prior to participation. The study including the consent procedure was approved by the local ethics committee (Re.-No. 3755). In total, 147 sera were analyzed, originating from 49 prostate cancer (PCa) patients, 70 benign prostatic hyperplasia (BPH) patients and 28 healthy male volunteers. Detailed information on all patients and healthy individuals is provided in Table 1.

**Table 1:** Detailed information on all patients and healthy individuals.

	PCa	BPH	Normal
**Number of Sera**	49	70	28
**Mean/Median age (years)**	67.3/67.5 (50.4–86.9)	69.3/69.4 (51.1–86.4)	55.8/55.9(50.8–64.7)
**PSA Mean/Median (ng/ml)**	9.59/7.26 (1.30–70.00)	4.4/2.4 (0.1–15.64)	
**Gleason sum**			
5	4		
6	12		
7	22		
8	2		
9	9		
**Grading**			
pT1a	2		
pT1b	3		
pT2a	7		
pT2b	1		
pT2c	17		
pT3a	12		
pT3b	6		
n. a.	1		

### Protein macroarray screening

We screened high-density protein macroarrays containing 38,016 *E*.*coli* expressed proteins of the hex1 (human fetal brain cDNA expression) library with pools of 150 sera of patients with different tumor and non-tumor diseases, including two pools of PCa sera (5 sera per pool) and one pool of 5 BPH sera.[22] A total of 1,827 clones, that were positive for autoantibodies in at least one serum pool, were selected and spotted in duplicates on subarray filters. These sub-macroarrays were screened with 49 PCa, 70 BPH and 28 normal sera (see S1 Fig).

In brief, macroarrays were washed two times in TBSTT (TBS, 0.05% Tween 20, 0.5% Triton X-100) and four times in TBS. After blocking with 3% non-fat dry milk in TBST (TBS, 0.05% Tween 20), macroarrays were incubated over night with diluted sera (1:1000). After incubation, sera were stored for a second round of incubation. The macroarrays were subjected to three washing steps with TBST followed by incubation with 70°C stripping solution. Afterwards macroarrays were washed two times in TBST and four times in TBS. After incubation with blocking solution macroarrays were subjected to the second round of serum incubation. Macroarrays were washed three times in TBST, and incubated with secondary antibody (rabbit anti-human IgG, IgA, IgM-Cy5 (H+L), Dianova, 1:1000). Finally, macroarrays were washed four times in TBST, two times in TBS and dried overnight. Signals were detected by scanning with Typhoon 9410 scanner (GE Healthcare).

### Image analysis and statistics

Spot intensity values were computed by ARCADIA, an image analysis software that we developed specifically for protein array evaluation [20]. In brief, the image of the macroarray was divided in subgrids. Those subgrids were further divided in spot areas containing one protein spot. Finally, the intensity of each spot was calculated as the mean value of all pixels of the respective protein spot.

We carried out standard quantile normalization to minimize array-to-array variations. Especially for features at the edge of the array we observed slightly decreasing quality. The respective features would however potentially bias the analysis. Thus, respective spots have been marked as not available. Next, we excluded 169 protein spots that were absent on more than 10 analyzed macroarrays. The remaining 1,658 clones were used for the classifications by a linear Support Vector Machine (SVM).

Altogether 20 repetitions of a standard 10-fold cross validation were performed and mean sensitivity, specificity, and accuracy for the four classification tasks was calculated. As a measure of the information content of single antigens for their ability to differentiate sera of two different groups area under the receiver-operator-characteristics-curve (ROC) values (AUC) were computed. The ROC curve shows the specificity as function of 1-sensitivity. For each antigen, all normalized intensity values for all sera were used as thresholds to discriminate patient sera from the controls. For all these thresholds, patient sera with intensity value above the threshold were considered as true positive (TP), patient sera with intensity value below the threshold as false negative (FN). Accordingly, control sera with intensity value below the threshold were considered as true negative (TN), control sera with intensity value above the threshold as false positive (FP). Subsequently, sensitivity (TP/(TP+FN)) and specificity (TN/(TN+FP)) of all thresholds were used to calculate ROC curve and AUC value of the considered antigen. If intensity values of the considered antigen in patient sera are higher than in control sera, AUC values range from 0 to 0.5. AUC values ranging from 0.5 to 1 confer to a higher mean intensity of the antigen in control sera compared to patient sera. We considered antigens with AUC values below 0.3 or above 0.7 as informative for the given classification. Finally, we computed the frequency of seroreactivity against the antigens in the considered groups by using a spot intensity threshold of 50 for positive seroreactivity. The protein macroarray data were deposited in the publicly available database Gene Expression Omnibus (GEO; http://www.ncbi.nlm.nih.gov/projects/geo/ website, GSE67068).

### Validation

In order to validate autoantibodies against immunogenic clones, we purchased respective clones from Source Bioscience, i.e., D02594, E19574, L16556, A22594, and C11549. Inserts were sequenced from 5’ and 3’ ends to confirm identity and length of the represented antigens. Sequence data revealed, that clone D02594 was a hybrid of the two proteins YBX1 and PDP1, representing at its 5’ end the first 275aa of YBX1 and at its 3’end the last 91aa (starting at aa446) of PDP1 with an unknown linker sequence in between. This protein was excluded from further analysis. The other 4 clones were confirmed to include sequences of RPS27A, RPL15, DDX54 and RPL7. To determine presence of autoantibodies against these antigens, we used the Xmap technology (Luminex, Austin, TX, USA). In short, his-tagged antigens were expressed in *E*.*coli* according to supplier’s recommendations, purified using Ni-NTA agarose beads and coupled to magnetic microspheres according to manufacturer’s instruction with minor modifications. Fifty μl of a beadmix containing 1000 coupled microspheres per assayed protein was incubated with sera of patients and controls (1:400 diluted in 100 mM Tris-HCl pH 7.0, 1%BSA 300 mM, NaCl 0.1%Tween-20) for 2h at RT on a shaker. Bound autoantibodies were detected using a 1:1 mixture of 2.5μg/ml R-Phyco AffiniPure F(ab')2 Frag Gt Anti-Hmn IgG [Fcγ] (Jackson ImmunoResearch Cat.No. 109-116-098) and 2.5μg/ml of R-Phyco AffiniPure F(ab')2 Frag Gt Anti-Hmn IgG [F(ab')2] (Jackson ImmunoResearch Cat.No. 109-116-097). After 1hr incubation with secondary antibodies, beads were washed three-times with PBS-Tween 0.05% and measured with Luminex Flexmap 3D. Besides standard hypothesis tests such as t-test or analysis of variance, the AUC has been calculated. Finally, the measurements were correlated to the Gleason score and PSA levels.

## Results

A total of 1,827 cDNA clones were screened with 147 serum samples, including 49 PCa sera, 70 BPH sera, and 28 sera from healthy men (Table 1). Out of these sera we selected an age-matched subset that included 44 PCa and BPH sera, each with a median age of 67 years. In addition we compared subsets of 36 sera from PCa patients with PSA level > 4 ng/ml and of 33 sera of BPH patients also with PSA level > 4 ng/ml.

### Frequency of immunogenic clones

**Fig 1:**
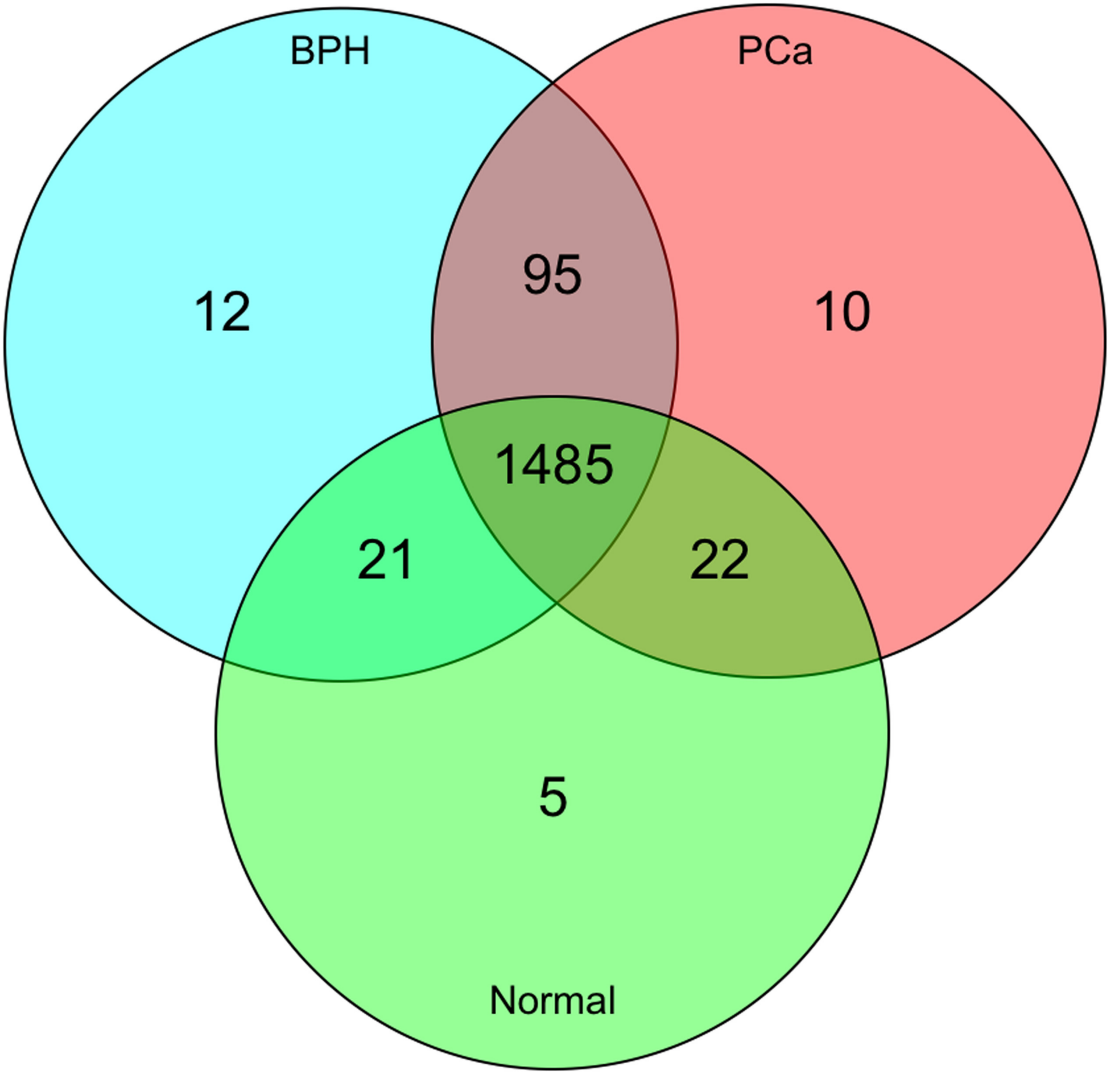
Distribution of the reacting clones. The Venn diagram shows the distribution of the 1658 reacting clones for the three serum groups (PCa, BPH, Normal). Most of the clones are reactive in all three groups whereas a small number of clones is just reactive in every single group.

To minimize array-to-array variations the 147 analyzed macroarrays were normalized using standard quantile normalization. We choose a spot intensity threshold of 50 for the determination of positive seroreactivity. We excluded all cDNA clones that showed lack of seroreactivity on more than 10 of the 147 arrays. Out of the remaining 1658 cDNA clones, 1612 clones reacted with at least one PCa serum. The frequency of seroreactivity varied from 2.3% for the cDNA clone the reacted least with the PCa sera, to 97.7% for the most reactive cDNA clone. We found 1613 clones that reacted with at least one BPH serum. The frequency of seroreactivity varied from 2.3% to 95.5%. Interestingly, 1,533 clones reacted with normal sera (frequencies from 3.6% to 96.4%. The comparison of the above mentioned clones revealed an overlap of 1,485 clones between PCa, BPH and normal sera. Those clones might be so called natural autoantibodies that are present in each serum of healthy persons at remarkable constant levels and characterized by minimal individual quantitative variations.[23] The Venn diagram in Fig 1 shows the distribution of the reacting clones.

As most of the analyzed antigens were reactive in sera of PCa patients, BPH patients, as well as healthy controls those clones seemed not to be very meaningful for further approaches. Therefore, we focused on those clones that only reacted with sera of one of the three serum groups, i.e., either PCa sera, or BPH sera or normal sera. Here we found ten clones only reactive with at least one PCa serum, 12 clones only reactive with at least one BPH serum, and 5 clones only reactive with at least one normal serum. Regarding the seroreactivity frequencies of each serum group the results were somewhat disappointing as at most only seven of 49 PCa sera reacted with the PCa specific antigens, at most only seven of the 70 BPH sera reacted with the BPH specific antigens, and only one normal serum reacted with the 5 specific antigens found only in normal sera. Therefore, it seems that these antigens might reflect individual variations.

### Information content of immunogenic clones

As considering only the seroreactivity frequencies per serum group seems not to be a good measure, we calculated for each cDNA clone the area under the receiver operator characteristics curve (AUC) as described in Materials and Methods section. The AUC is a measure for the information content of each antigen for the separation of two different serum groups from each other. Here it became evident, that clones that react with more than one serum group can, however, have a high information content for certain separations.

The AUC values of the 1,658 analyzed clones, including 461 in-frame clones, are summarized in Table 2. We ranked the cDNA clones according to the information content they contributed to the different classification tasks. Clones with an AUC value > 0.7 or < 0.3 were considered to be very informative for a given classification task.

**Table 2:** Distribution of clones according to the AUC values that were determined for the classification of PCa vs. Normal, BPH vs. Normal, PCa vs. BPH, and PCa (PSA>4.0) vs. BPH (PSA>4.0).

	PCA vs. Normal		BPH vs. Normal		PCA vs. BPH		PCa (PSA>4.0) vs. BPH (PSA>4.0)	
AUC value	N^o.^ (all)	N^o.^ (in-frame)	N^o.^ (all)	N^o.^ (in-frame)	N^o.^ (all)	N^o.^ (in-frame)	N^o.^ (all)	N^o.^ (in-frame)
0.0–0.1	0	0	0	0	0	0	0	0
0.1–0.2	7	0	5	1	0	0	4	2
0.2–0.3	114	20	66	5	5	2	77	26
0.3–0.4	263	65	266	40	187	74	270	80
0.4–0.5	443	117	536	135	642	205	473	156
0.5–0.6	484	145	531	178	588	133	453	113
0.6–0.7	269	82	215	84	223	45	290	70
0.7–0.8	76	31	39	18	13	2	82	12
0.8–0.9	2	1	0	0	0	0	9	2
sum	1658	461	1658	461	1658	461	1658	461

For the discrimination of PCa and normal sera, 199 clones (12%) were considered to be informative, including 52 in-frame clones. Among the informative clones, 78 clones showed AUC values > 0.7, including 32 in-frame clones and 121 clones showed AUC values < 0.3, including 20 in-frame clones. The out-of-frame clone D20552 with a sequence homology to the brain creatine kinase (CKB) showed the lowest AUC value (AUC = 0.142) for the discrimination of PCa and normal sera. This clone was also informative for the discrimination of BPH and normal sera (AUC = 0.190) but not for the discrimination of PCa and BPH sera (AUC = 0.466). The out-of-frame clone E03526 with sequence homology to dimethylarginine dimethylaminohydrolase 1 (DDAH1) showed the highest AUC value (AUC = 0.811). Again, this clone was also informative for the discrimination of BPH and normal sera (AUC = 0.760) but not for the discrimination of PCa and BPH sera (AUC = 0.558).

The clone D02594 with sequence homology to nuclease sensitive element binding protein 1 (YBX1) was the in-frame clone with the lowest AUC value (AUC = 0.209) and the clone A06579 with sequence homology to protein (peptidyl-prolyl cis/trans isomerase) (PIN4) was the in-frame clone with the highest AUC value (AUC = 0.806).

For the discrimination of BPH and normal sera, 110 clones (6.63%), including 24 in-frame clones were considered to be informative. Among them, 39 clones showed AUC values > 0.7, including 5 in-frame clones and 71 clones showed AUC values < 0.3, including 6 in-frame clones. The out-of-frame clone C18596 showed the lowest AUC value (AUC = 0.144) for the discrimination of BPH and normal sera, i.e., this clone showed a stronger immune response with sera of BPH patients compared to normal sera. This clone was also informative for the discrimination of PCa and normal sera (AUC = 0.236) and showed stronger immune response in sera of PCa patients compared to normal sera. However, for the discrimination of BPH sera and PCa sera the clone C18596 reached only an AUC value of 0.528 and had therefore less information content for this separation. The out-of-frame clone E13519 with a sequence homology to oxidase (cytochrome c) assembly 1-like (OXA1L) showed the highest AUC value (AUC = 0.782) specific for the discrimination of BPH and normal sera, i.e., higher immunogenicity in normal sera than in BPH sera. However, this clone had no information content for the separation of PCa from normal sera (AUC = 0.646) and for the separation of BPH from PCa (AUC = 0.446). For the discrimination of BPH and normal sera the clone E19574 with sequence homology to the pseudogene similar to ubiquitin and ribosomal protein S27a precursor was identified as the most informative in-frame clone with AUC < 0.3, i.e., higher immune response in sera of BPH patients than in normal sera (AUC = 0.175). The in-frame clone L16556 with sequence homology to ribosomal protein L15 (RPL15) was identified as the most informative in-frame clone with AUC > 0.7, i.e., higher immunogenicity in normal sera compared to sera of BPH patients (AUC = 0.773).

When comparing PCa and BPH sera, 99.3% (1646 clones) of the immunogenic clones showed AUC values between 0.3 and 0.7, which means they are non-informative. Only 18 informative clones (1.09%), including four in-frame clones, remained. Among them, 5 clones showed AUC values < 0.3 and 13 clones showed AUC values > 0.7. The out-of-frame clone N22510 with a sequence homology to MARCKS-like 1 (MARCKSL1) showed the highest AUC value (AUC = 0.773) for the discrimination of PCa and BPH. The clone A22594 showed the lowest AUC value (AUC = 0.266). Both clones were also informative for the discrimination of BPH and normal sera, but not for PCa sera vs. normal sera. The latter clone A22594 was in-frame and thereby the clone with the lowest AUC. The clone C11549 with sequence homology to 60S ribosomal protein L7 was the in-frame clone with the highest AUC value (AUC = 0.710).

For the discrimination of sera from patients with different diseases of the same organ, i.e., PCa or BPH, we also took the pre-operative PSA value into account. Therefore, we selected 36 PCa sera and 33 BPH sera with PSA ≥ 4.0 ng/ml from the initial study population. The overlap to the before mentioned age-matched serum cohort was 32 PCa and 17 BPH sera. The following classification revealed 166 informative clones, including 42 in-frame clones. Among the informative clones, 81 clones showed AUC values < 0.3, including 28 in-frame clones and 91 clones showed AUC values > 0.7, including 14 in-frame clones. In the discrimination PCa versus BPH, each with PSA ≥ 4.0 ng/ml, the out-of-frame clones N12603 and N22510 showed the best AUC values of 0.174 and 0.838, respectively. Clone N12603 has sequence homology to cholinergic receptor nicotinic alpha (CHRNA2) and clone N22510 to MARCKS-like 1 (MARCKSL1). Interestingly, the latter clone N22510 was also the clone with the highest AUC value for the separation PCa versus BPH, independent of the PSA level.

The clones A18602 (sequence homology to ribosomal protein S2 (RPS2)) and J04520 (sequence homology to Y box binding protein 1 (YBX1)) were the in-frame clones with the best AUC values of 0.188 and 0.818, respectively.

### Classification results

As detailed in the Materials and Methods section, classification was carried out by a linear Support Vector Machine (SVM). Here, 20 repetitions of a standard 10-fold cross validation were performed and mean sensitivity, specificity, and accuracy for four classification tasks was calculated. All classification results are detailed in Table 3. The best results were obtained for the classification BPH versus normal with an accuracy of 86%. The classification PCa versus normal yielded an accuracy of 83.2%. The worst classification accuracy of 70.3% was obtained for the age matched samples PCa versus BPH. But, considering the PSA level of > 4.0 clearly improved the classification results. Here we obtained an accuracy of 84.1%.

**Table 3:** Mean values of Accuracy, Specificity and Sensitivity for the classifications of PCa vs. Normal, BPH vs. Normal, PCa vs. BPH, and PCa (PSA>4.0) vs. BPH (PSA>4.0).

Classification	Accuracy	Specificity	Sensitivity
**PCa (n = 44) vs. Normal (n = 28)**	83.2% (82.4–84.0)	91.5% (90.4–92.6)	70.2% (69.0–71.3)
**Random**	52.3% (49.6–54.9)	63.4% (60.2–66.6)	34.8% (31.5–38.2)
**BPH (n = 44) vs. Normal (n = 28)**	86.0% (85.3–86.7)	93.6% (92.5–94.8)	73.9% (72.5–75.4)
**Random**	51.9% (49.1–54.8)	64.3% (61.5–67.1)	32.5% (27.9–37.1)
**PCa (n = 44) vs. BPH (n = 44) age matched**	70.3% (68.6–72.1)	67.5% (64.9–70.1)	73.2% (71.1–75.2)
**Random**	49.8% (47.6–52.0)	49.3% (46.5–52.1)	50.3% (47.9–52.8)
**PCa (PSA>4.0, n = 36) vs. BPH (PSA>4.0, n = 33)**	84.1% (82.8–85.5)	83.8% (82.2–85.3)	84.5% (82.9–86.2)
**Random**	48.8% (45.8–51.7)	51.3% (47.5–55.0)	46.1% (42.2–49.9)

### Validation

Since high-throughput technologies frequently lead to false positive biomarker results, validation by independent technology is essential. From the previous analyses we picked five clones: D02594, E19574, L16556, A22594, and C11549. These correspond to proteins RPS27A, RPL15, DDX54 and RPL7. Since re-sequencing demonstrated that clone D02594 was a hybrid of the two proteins YBX1 and PDP1, it was left out from the statistical analysis. The clones were screened with a cohort of 84 individuals, including 27 PCa patients, 30 BPH patients and 27 controls. An analysis of variance showed significant results for DDX54 (p = 0.03) and RPL7 (p = 0.04). A third clone, RPL15, slightly exceeded the significance threshold (p = 0.074).

**Fig 2:**
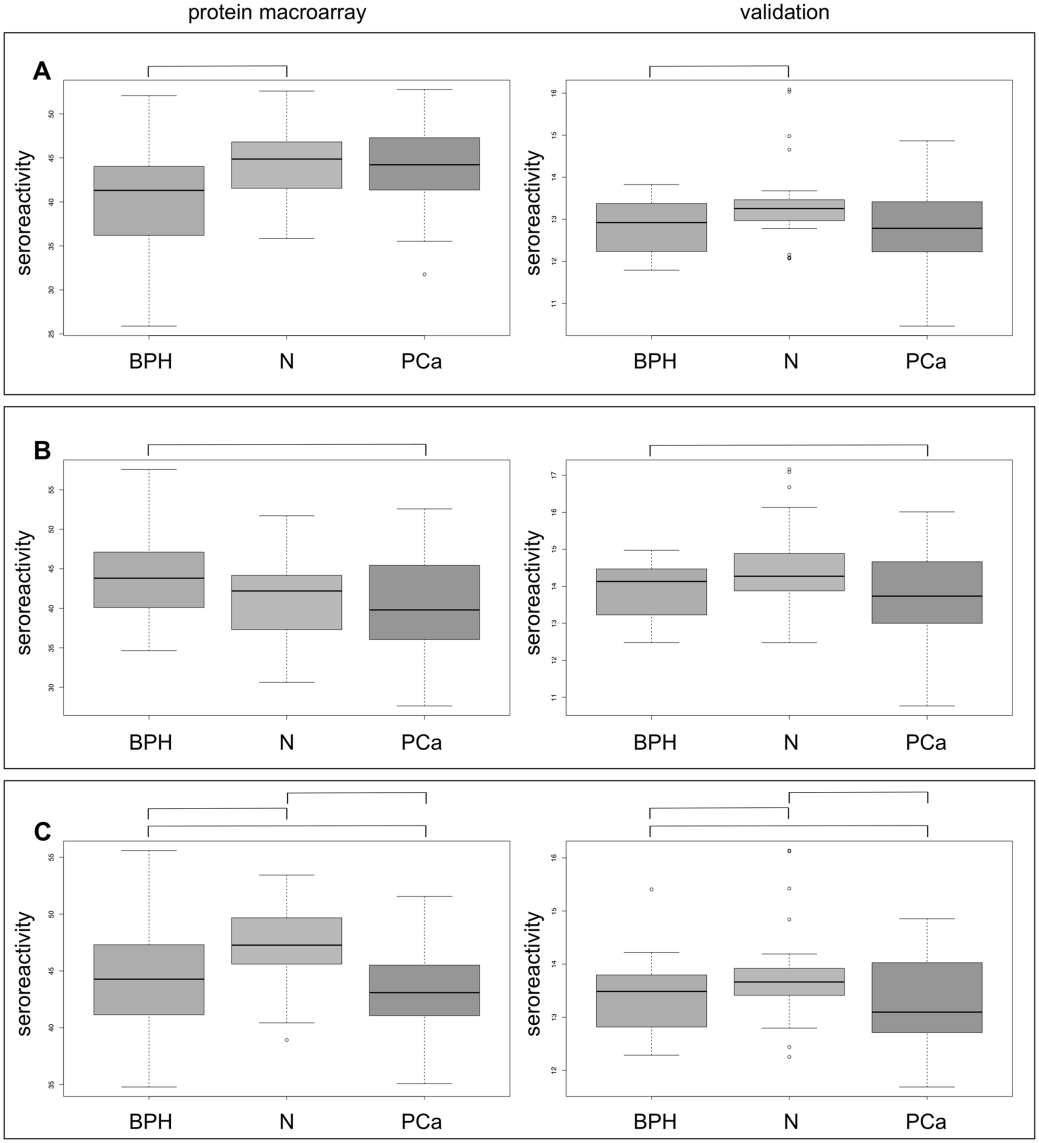
Seroreactivity against DDX54, RPL7 and RPL15 in protein macroarray and Luminex validation. Box-Whisker plots for reactivity against DDX54 (A), RPL7 (B) and RPL15 (C) with serum of patients with benign prostate hyperplasia (BPH), prostate carcinoma (PCa) and healthy controls (N) in protein macroarray (left panels) and luminex validation (right panels). For each group, the boxes indicate the 2. and 3. quartile of seroreactivity, the whiskers show minimum and maximum value. The horizontal black lines indicate the median seroreactivity in the group. Similar seroreactivity trends are indicated by square brackets.

In the screening experiments, DDX54 showed an increased median seroreactivity in control sera (44.9) and PCa patients (44.6) compared to BPH (41.2) patients. This higher reactivity in controls compared to BPH patients has been confirmed by the validation analysis (Fig 2A). For the second protein, RPL7, the initial results increased autoantibodies in BPH patients (44.8) compared to PCa patients (39.0). This trend was also observed in the validation experiments (Fig 2B). Although slightly not statistically significant, the overall trend of higher seroreactivity against RPL15 in normal samples compared to both PCa and BPH patients was successfully reproduced in validation (Fig 2C). By correlating the protein expression to the PSA levels we did not observed significant results.

## Discussion

There is common consensus that PSA screening has decreased the mortality rate of PCa patients for years. Nevertheless, PSA testing is still a contentious issue for it often leads to false results, overdiagnosis, and false therapy going along with high costs.[24] The identification of further biomarkers, e.g., PCA3, GSTP1, AMACR, or miRNAs, reveals the opportunity to improve prostate cancer detection.[25] Additionally, new biomarkers may enhance monitoring and give a more detailed view into cancer progress and development.

In our study, we present a combination of autoantibody signature with PSA level. We screened a set of 1,827 cDNA clones with 49 PCa sera, 70 BPH sera, and 28 sera from healthy individuals by protein macroarray analysis. Additionally, we compared seroactivity of PCa patients, BPH patients and controls by calculating AUC values. AUC values < 0.3 and > 0.7 were considered as informative. In total, we identified 199 informative clones (including 52 in-frame-clones) for the classification of PCa versus normal, 110 informative clones (including 24 in-frame-clones) for the classification of BPH versus normal, and only 18 informative clones (including 4 in-frame-clones) for the classification of PCa versus BPH. Considering only PCa and BPH sera with PSA levels > 4.0 ng/ml we detected 166 clones (including 42 in-frame-clones) for the classification of PCa versus BPH.

We were able to separate sera from PCa patients and controls with an accuracy of 83.2%, a sensitivity of 70.2%, and a specificity of 91.5%. BPH sera and normal sera were separated with an accuracy of 86.0%, a sensitivity of 73.9%, and a specificity of 93.6%. The worst classification accuracy with 70.3% was obtained for PCa versus BPH. Interestingly, the classification result improves after taken the PSA level of > 4.0 ng/ml into consideration. Here, we separated PCa sera and BPH sera with an accuracy of 84.1%, a sensitivity of 84.5%, and a specificity of 83.8%. This is of importance because this method allows separating malignant disease from benign disease.

Our results are based on specific reactions of the immune system. Beside PSA and other biomarkers cancer-associated antigens may be very specific marker and suitable as diagnostic tool. The immune system recognizes cancer cells by producing specific autoantibodies against these cancer-associated antigens. Autoantibodies can be easily detected in patients`sera. Moreover, they have a long half-life and are detectable at low costs.[26] The identified antigens detected in the sera of PCa patients and BPH patients may serve as biomarkers or as targets for future therapy for patients with different prostate diseases. The antigens we found may play a functional role in the development of prostate cancer. In total, we found 12 phage-peptide clones with homology to known proteins. Clones with homology to CKB, DDAH1, YBX1, PIN4, OXA1L, pseudogene similar to ubiquitin and ribosomal protein S27a precursor, and RPL15 were informative for the discrimination of normal sera and PCa and BPH, respectively. Clones with homology to 60S ribosomal protein L7, and MARCKS1 were informative for the discrimination of PCa sera and BPH sera. Clones with homology to CHRNA2, RPS2, YBX1, and MARCKS1 were informative for the discrimination of PCa sera and BPH sera taken the PSA level into consideration. Interestingly, the phage-peptide clone with a homolog sequence to MARCKSL1 was very informative for the classification of PCa versus BPH with and without considering the PSA level. MARCKSL1 is a member of the MARCKS family, a group of proteins involved in the calmodulin (CaM) signaling pathway, the protein kinase C (PKC) signaling pathway, and in the regulation of the actin cytoskeleton.[27] MARCKS can be associated with cancer development and tumorgenesis.[28] MARCKS was previously been reported to be a target of miRNA-21 in prostate cancer cells.[29] Further studies have to elucidate the exact role of MARCKS in the development of prostate cancer. It may be exploited as future diagnostic tool to distinguish between malignant state and benign state.

In addition, further phage-peptide clones with homology to YBX1, RPL15 and CHRNA2 have been associated with cancer and other disease. YBX1 has been associated with several human cancers, e.g., colorectal cancer, breast cancer, and glioblastome multiforme.[30–32] RPL15 is overexpressed and seems to be involved in cell proliferation in gastric cancer.[33] CHRNA2 is located on chromosome 8p, a chromosomal region with 484 detected genes. Several of these genes are supposed to be tumor-suppressor genes or oncogenes.[34] Future studies have to disclose their role in prostate cancer progress and development. In our study, we provide evidence that blood testing discriminates PCa and BPH patients’ sera from control sera with high specificity and sensitivity.

Since high-throughput screening approaches frequently lead to false positive biomarker candidates, validation using other technologies is required to find true positives. We thus carried out validation experiments on 84 samples on five proteins. One of these proteins was excluded since the original sequence did not match the control sequencing and corresponded to a chimeric protein (YBX1/PDP1). For another protein, RPS27A, we were not able to find differential seroreactivity in the three cohorts. With respect to the remaining three proteins differential seroreactivity was confirmed by the experiments and just in one cases we observed a different pattern, a lower seroreactivity for PCa patients. The dys-regulation is driven by four patients with high seroreactivity and four patients with low seroreactivity, demonstrating that individual measurements have a substantial influence of the pattern, especially if the cohort size is limited. Given the present cohort sizes and the screening approach, the validation matched the original results in many cases but also identified, as expected, false positive markers.

Moreover, our results of a combined autoantibody screening along with PSA testing allow a high discrimination of PCa patients and BPH patients. The results may pave the way for a new diagnostic tool that enables characteristic patterns for patients with PCa and BPH respectively. Although prostate biopsy is favored as a histological diagnosis of prostate cancer for patients with a PSA level greater than 4 ng/ml, in almost one of two patients symptoms occur caused by biopsy, like fever, haematuria, haematochezia or haemoejaculate and often results in loss of toleration for repeating biopsy.[2, 35] Moreover, diagnostic methods, which are able to distinguish between malignant state and benign state, also reduce the risk of patients`undertreatment and overtreatment.

## Supporting Information

S1 Fig General Workflow of the Protein macroarray screening. (TIF)
