# Supplementary figures and images for: Combination of Autoantibody Signature with PSA Level Enables a Highly Accurate Blood-Based Differentiation of Prostate Cancer Patients from Patients with Benign Prostatic Hyperplasia
Source: PLoS One. 2015 Jun 3;10(6):e0128235. doi: 10.1371/journal.pone.0128235 (PMC4454546; doi:10.1371/journal.pone.0128235)

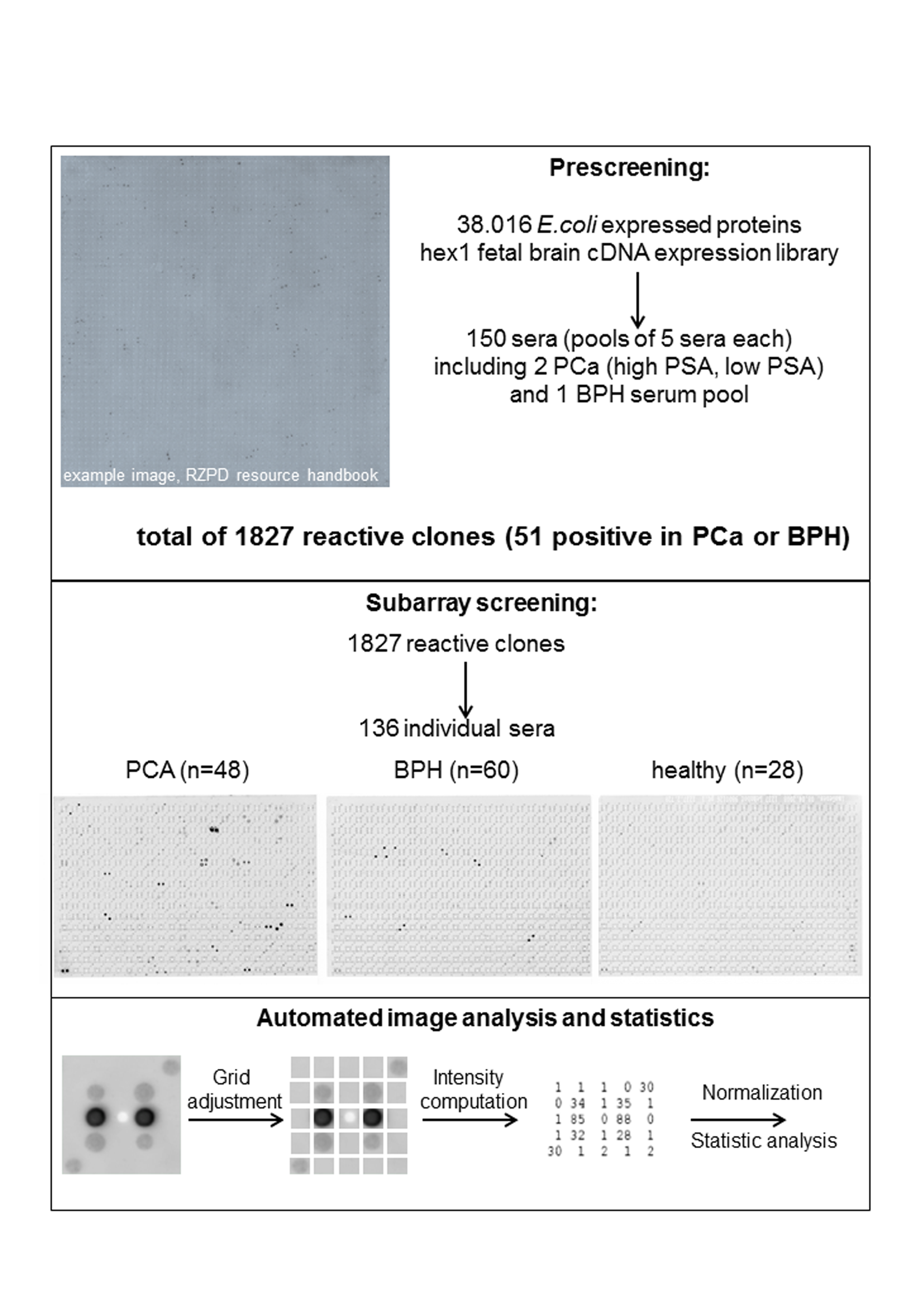

Supplement: S1 Fig — (TIF) [file pone.0128235.s001.tif]
